# Impact of reduced uterine perfusion pressure model of preeclampsia on metabolism of placenta, maternal and fetal hearts

**DOI:** 10.1038/s41598-022-05120-2

**Published:** 2022-01-21

**Authors:** Lana McClements, Claire Richards, Nikayla Patel, Hao Chen, Kimberly Sesperez, Kristen J. Bubb, Anja Karlstaedt, Dunja Aksentijevic

**Affiliations:** 1 School of Life Sciences, Faculty of Science, University of Technology Sydney, Sydney, NSW Australia; 2 Centre for Biochemical Pharmacology, William Harvey Research Institute, Barts and the London School of Medicine and Dentistry, Queen Mary University of London, Charterhouse Square, London, EC1M 6BQ UK; 3 Biomedical Discovery Institute, Monash University, Melbourne, Australia; 4 Department of Cardiology, Smidt Heart Institute, Cedars Sinai Medical Center, Los Angeles, CA USA 127 San Vincente Blvd, 90048

**Keywords:** Metabolism, Reproductive biology, Cardiovascular biology, Heart development, Metabolomics, Hypertension, Biochemistry, Developmental biology, Cardiology, Diseases

## Abstract

Preeclampsia is a cardiovascular pregnancy complication characterised by new onset hypertension and organ damage or intrauterine growth restriction. It is one of the leading causes of maternal and fetal mortality in pregnancy globally. Short of pre-term delivery of the fetus and placenta, treatment options are limited. Consequently, preeclampsia leads to increased cardiovascular disease risk in both mothers and offspring later in life. Here we aim to examine the impact of the reduced uterine perfusion pressure (RUPP) rat model of preeclampsia on the maternal cardiovascular system, placental and fetal heart metabolism. The surgical RUPP model was induced in pregnant rats by applying silver clips around the aorta and uterine arteries on gestational day 14, resulting in ~ 40% uterine blood flow reduction. The experiment was terminated on gestational day 19 and metabolomic profile of placentae, maternal and fetal hearts analysed using high-resolution ^1^H NMR spectroscopy. Impairment of uterine perfusion in RUPP rats caused placental and cardiac hypoxia and a series of metabolic adaptations: altered energetics, carbohydrate, lipid and amino acid metabolism of placentae and maternal hearts. Comparatively, the fetal metabolic phenotype was mildly affected. Nevertheless, long-term effects of these changes in both mothers and the offspring should be investigated further in the future.

## Introduction

Cardiovascular disease is the main cause of death worldwide. Converging lines of evidence suggest that an adverse intrauterine environment precipitates maternal, placental and fetal adaptations, resulting in the developmental programming of cardiometabolic dysfunction in juvenile and adult offspring^1–3^. Preeclampsia is a cardiovascular pregnancy complication that occurs in 5–10% of pregnancies and is one of the leading causes of maternal and fetal morbidity and mortality worldwide^4^. It is associated with poor pregnancy outcomes and can lead to lifelong risk from cardiovascular complications in the offspring: an early onset of cardiac hypertrophy, aortic remodelling^5–8^, oxidative stress, endothelial dysfunction, hypertension, and adverse ischemia–reperfusion (IR) outcomes in adult life^2^. Compared to normotensive pregnancies, mothers with early-onset preeclampsia presenting prior to 34 weeks of gestation (EOPE) have increased mortality risk (up to 9–10-fold) from cardiovascular disease, whereas mothers diagnosed with late-onset preeclampsia after 34 weeks gestation (LOPE) are at a two-fold increased risk of CVD-related mortality^9,10^. Recently, multiple common features were recognized between preeclampsia and heart failure with preserved ejection fraction (HFpEF) including factors such as hypertrophy, angiogenesis, inflammation and haemostasis^11,12^. However, the exact metabolic adaptations, which may impact in utero development and may lead to cardiometabolic consequences in adult life, have not been fully elucidated. Moreover, effective therapeutic and diagnostic strategies are still missing due to the lack of mechanistic insight into the causal nexus of preeclampsia. Nevertheless, an inability of the maternal cardiovascular system to alleviate preeclampsia-induced changes appears to have a critical role in defective placental development including oxidative stress, abnormal angiogenesis and pro-inflammatory responses^13^.

Pre-clinical models of preeclampsia are challenging to establish, due to the presence of inter-species variations and the fact that most animals do not develop spontaneous preeclampsia^14^. The most reliable animal model of preeclampsia is the surgically-induced reduced uterine perfusion pressure (RUPP) model. This model has been shown to induce hypertension^15^, proteinuria^15^, renal dysfunction^15,16^, an anti-angiogenic state^17^, inflammation^18–20^, vasoconstriction^21^, oxidative stress^22^, cardiac dysfunction^23^ and intrauterine growth restriction (IUGR)^24,25^ like that of preeclampsia in humans.

We have also recently shown in this model increased cardiac expression of the anti-angiogenic protein, FKBPL, and the presence of placental and cardiac fibrosis^26^. Given the significant association between preeclampsia and CVD, a more thorough investigation of cardiac health in the RUPP model is required. In this study, we demonstrate in the RUPP rat model of preeclampsia, the presence of cardiovascular and metabolic remodelling of maternal heart and placenta. We also show that the series of functional and metabolic maternal adaptations to early restricted angiogenesis precede metabolic alterations in the fetal heart.

## Methods

### Reduced uterine perfusion pressure pregnancy model in rats

Time-mated pregnant Sprague–Dawley rats were purchased from the Animal Resources Centre (ARC) and were fed a standard sterile chow diet with water access ad libitum. On gestational day (GD) 14, pregnant rats were randomised to the Sham (control; n = 8) or RUPP (n = 7) procedure as previously described^15,26,27^. Briefly, silver clips were applied to the aorta above the iliac bifurcation (0.203 mm ID) and both the right and left uterine arcades (0.100 mm ID) to reduce the blood flow to the uterus by ~ 40% in the RUPP group whereas rats in the Sham (control) group underwent a similar procedure, without clips being applied. On GD19, after clinical phenotyping was completed, rats were euthanized as per local standard operating procedures using isoflurane overdose/exsanguination followed by removal of the heart. Maternal hearts, placentae and embryo hearts were collected, weighed and snap frozen in liquid N_2_. All clinical phenotyping, placental and embryo data for these rats were reflective of the preeclampsia phenotype as published previously^26^ and reproduced here with permission (Table 1). For this study, five matching maternal hearts, placenta and fetal hearts were available from each group for downstream metabolomic analysis as described below. All animal experiments were approved by the Northern Sydney Local Health District Animal Ethics Committee (Animal Ethics number: RESP/18/317) and carried out in accordance to the Australian code for the care and use of animals for scientific purposes and ARRIVE guidelines.

**Table 1:** Morphological features and cardiac function characteristics of the RUPP model (maternal and fetal).

	Sham (*n* ≥ 6)	RUPP (*n* ≥ 4)	*P* value
Maternal body weight (before surgery), g	338.6 ± 17.5	379.5 ± 4.87	0.084
Maternal heart weight, g	1.02 ± 0.05	1.22 ± 0.01	0.008**
Heart: Body weight, %	0.304 ± 0.01	0.323 ± 0.005	0.303
Embryo resorption (%)	2.38 ± 1.51	11.22 ± 3.06	0.04*
Embryo weight, g	1.68 ± 0.025	1.61 ± 0.02	**0.04**
Placental weight, g	0.41 ± 0.009	0.39 ± 0.006	**0.047**
Heart rate, bpm	369.3 ± 4.8	398.8 ± 3.7	0.0006***
Systolic BP, mmHg	112.6 ± 1.3	127.8 ± 1.9	< 0.0001***
Diastolic BP, mmHg	87.6 ± 1.7	104.0 ± 1.8	< 0.0001***
MABP, mmHg	100.9 ± 1.5	116.7 ± 1.7	< 0.0001***
Stroke volume, µl	215.0 ± 4.326	230.0 ± 13.26	0.304
Cardiac output, mL/min	79 ± 3	85 ± 5	0.067
Ejection fraction, %	82 ± 2	78 ± 2	0.233
Fractional shortening, %	52 ± 2	49 ± 2	0.236
Corrected LV mass, mg	600 ± 14	706 ± 68	0.037*
LV anterior wall systolic, mm	2.9 ± 0.12	2.9 ± 0.11	0.672
LV anterior wall diastolic, mm	1.5 ± 0.04	1.8 ± 0.17	0.152
LV posterior wall systolic, mm	2.8 ± 0.14	2.9 ± 0.09	0.303
LV posterior wall diastolic, mm	1.6 ± 0.05	1.6 ± 0.11	0.994

Unpaired Student’s t-test or Mann–Whitney test were used depending on data distribution.

This figure was originally published in Biology of Sex Differences and used with permission^26^ (https://doi.org/10.1186/s13293-021-00376-1).

*BP* blood pressure, *LV* left ventricular, *MABP* mean arterial blood pressure, *RUPP* reduced uterine perfusion pressure.

*P < 0.05, ***P < 0.001.

**P < 0.01

### ^1^H Nuclear magnetic resonance spectroscopy metabolomic profiling

Frozen, weighed and pulverized hearts (fetal and maternal) and placentae were subjected to methanol/water/chloroform dual-phase extraction^28^. The aqueous upper phase was separated from the chloroform and protein fractions. 30 mg chelex-100 was added to chelate paramagnetic ions, vortexed and centrifuged at 3600 RPM for 5 min at 4 °C. The supernatant was added to a fresh Falcon tube containing 10 µL universal pH indicator solution followed by vortexing and lyophilisation. Dual-phase-extracted metabolites were reconstituted in 600 µL deuterium oxide (containing 8 g/L NaCl, 0.2 g/L KCl, 1.15 g/L Na2HPO4, 0.2 g/L KH2PO4 and 0.0075% w/v trimethylsilyl propanoic acid (TSP)) and adjusted to pH ≈ 6.5 using 1 M hydrochloric acid and/or 1 M sodium hydroxide^28^.

Samples were analysed using a vertical-bore, ultra-shielded Bruker 14.1.T (600 MHz) spectrometer with a bbo probe at 303 K. Spectra were acquired with the Bruker noesygppr1d pulse sequence with 128 scans, 4 dummy scans and 20 ppm sweep width, acquisition time of 2.6 s, pre-scan delay of 4 s, 90° flip angle and experiment duration of 14.4 min. TopSpin (version 4.0.5) software was used for data acquisition and for metabolite quantification. FIDs were multiplied by a line broadening factor of 0.3 Hz and Fourier-transformed, phase and automatic baseline-correction were applied. Chemical shifts were normalized by setting the TSP signal to 0 ppm. In terms of glycogen quantification, given that it is a large macromolecule containing multiple glucose monomers, we measured a number of mobile ^1^H in the glucose monomers that are present in the NMR peak, normalized to the reference.

Peaks of interest were integrated automatically using a pre-written integration region text file and then manually adjusted where required. Assignment of metabolites to their respective peaks was carried out based on previously obtained in-house data, confirmed by chemical shift and using Chenomx NMR Profiler Version 8.1 (Chenomx, Canada). Peak areas were normalized to the TSP peaks and metabolite concentrations were quantified per gram tissue wet weight^29,30^.

### Real-time quantitative polymerase chain reaction (RT-qPCR)

Relative mRNA expression of *galectin-3* in the placentae in Sham and RUPP rats was measured by RT-qPCR. Frozen tissue was homogenised with TRIsure reagent (Bioline, Australia) and isolated according to the manufacturer’s protocol. Once purified, the RNA was reverse transcribed to cDNA using a Tetro cDNA synthesis kit (Bioline, Australia) and relevant primers (Supplemental Table S1). RT-qPCR was performed using a SensiFAST SYBR No-ROX kit (Bioline, Australia) according to the manufacturer’s protocols and CFX96 Real-Time System thermal cycler (Bio-Rad, United States). The mRNA expression levels were normalized to those of *β-actin* and transformed by the ΔΔCT method.

### Western blotting

Protein lysates were generated from frozen tissue samples by homogenising with RIPA lysis buffer. Protein was quantified by BCA assay (Thermo Fisher, United States) prior to separation by SDS-PAGE. Following transfer, the membranes were imaged for total protein using the Stain Free imaging component of the ChemiDoc imaging system (Bio-Rad, United States) and blocked with 5% skimmed milk. The membranes were probed with mouse anti-HIF1-α monoclonal antibody (BD Bioscience, United States) and corresponding sheep HRP-linked anti-mouse IgG secondary antibody (GE Healthcare, United Kingdom). Immunoreactive bands were visualised by reacting with Clarity Western ECL (Bio-Rad, United States) and imaged using the ChemiDoc. Relative HIF1-α protein expression was normalized to the intensity of bands detected in the stain free image of each blot as a loading control.

### Metabolic network analysis of placenta

Given that the most extensive metabolite concentration changes were identified in placenta (> 2 metabolites), network plots of metabolite co-regulation were generated with the igraph package in R programming language (4.1.0). Each node was representative of a significantly different metabolite from different tissues in RUPP mice compared to the sham controls. Only the strongly correlated nodes were shown (absolute Pearson correlation coefficient $\left| {\text{r}} \right| > 0.7$), and colour of each edge was indicative of Pearson r. The functional classifications of nodes were annotated as the colour of nodes. Each node was sized proportionally to the epigenvector centrality (the importance of the network).

### In Silico modelling

In silico simulations (maternal hearts) were conducted using the metabolic network of the cardiomyocyte CardioNet^31,32^ as described in the Supplementary Information. Details of all reactions and their metabolic subsystems were annotated based on the Kyoto Encyclopedia of Genes and Genomes database^33^.

### Statistical analysis

Statistical analysis was performed in GraphPad Prism (v9). Normality of data distribution was examined using Shapiro–Wilk’s normality test. Sham and RUPP groups were compared using the unpaired student’s t-test (normally distributed data) and Mann–Whitney U test (non-normally distributed data) with statistically significant results equivalent to a p-value of 0.05 or less.

## Results

**Figure 1:**
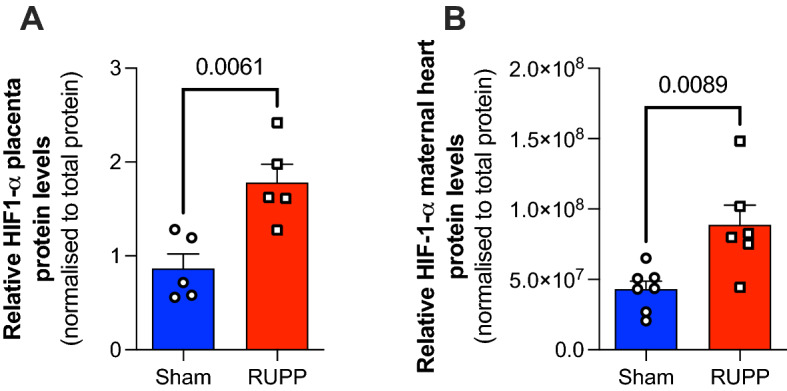
RUPP increases HIF1α expression in placenta and maternal hearts. RUPP leads to increase in HIF-1α protein expression in placenta (**A**) and maternal hearts (**B**). Protein lysates were extracted from placenta and heart from each rat and western blotting performed to determine the expression of HIF-1α relative to the total protein. Two group comparison was carried out using the student t test, n ≥ 5; *P* values < 0.05 shown in individual plots.

The pre-clinical rat model of preeclampsia resulting from the RUPP surgical intervention on gestational day 14 led to a series of maternal and fetal physiological adaptations (Table 1). We have recently shown that the RUPP procedure leads to reduced placenta and fetal weight as well as increased fetal resorption (Table 1)^26^. Furthermore, it triggers cardiac and placental fibrosis, maternal haemodynamic overload resulting in increased heart rate, systolic, diastolic and mean arterial pressures collectively resulting in increased LV mass and cardiac hypertrophy indicative of diastolic dysfunction (Table 1)^26^. However, there was no evidence of maternal cardiac failure as in vivo cardiac function parameters including ejection fraction, cardiac output and stroke volume were comparable to sham controls^26^. These observations are collectively reflective of preeclampsia in humans. Furthermore, RUPP intervention leads to placental hypoxia indicated by increase in hypoxia inducible factor (HIF)-1α protein expression (Fig. 1; *P* < 0.05). However, there were no changes in the mRNA expression of the inflammation marker galactin-3 in maternal tissues (placenta and heart, Supplementary Fig. S1).

### RUPP impact on placental, maternal and fetal cardiac metabolism

**Figure 2:**
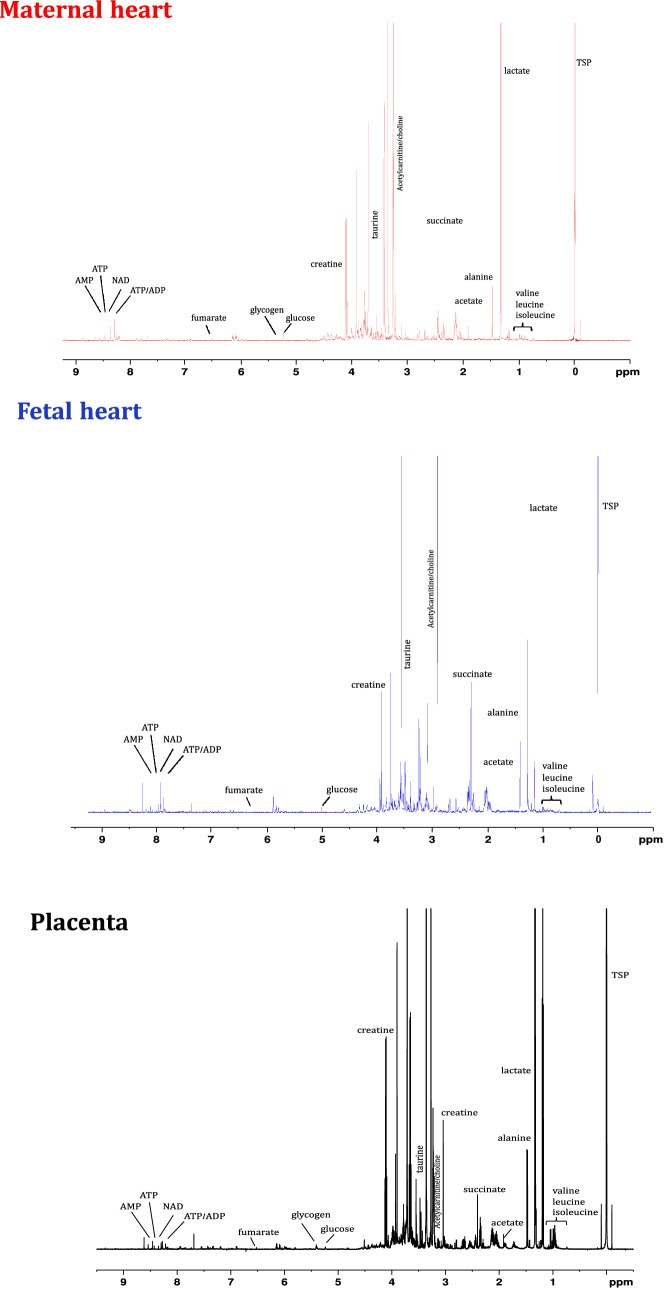
Representative ^1^H NMR spectra from heart (maternal and fetal) and placenta.

**Figure 3:**
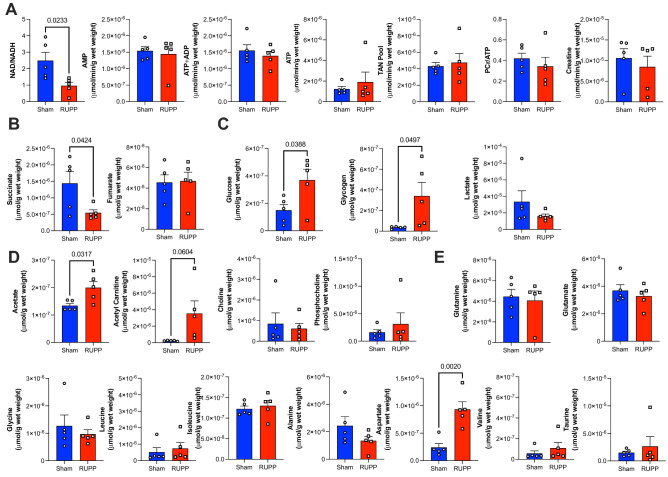
Metabolomic profile of RUPP model of maternal hearts. Metabolite concentrations are grouped into (**A**) Redox and Energetics (**B**) TCA cycle (**C**) Carbohydrate metabolism (**D**) Lipid metabolism (**E**) Amino acid metabolism. Two group comparison was carried out using the student T test (normally distributed data) or Mann-Whitney U test (non-normally distributed data: acetate, aspartate, NADH, lactate, leucine, valine, lactate, glutamine, choline) n = 5/group. TAN-total adenine nucleotide pool (AMP + ADP + ATP). PCr-phosphocreatine n ≥ 5; *P* values < 0.05 shown in individual plots.

Using high resolution ^1^H NMR spectroscopy (representative spectra shown in Fig. 2) we were able to identify a series of changes in the metabolomic profiles of maternal hearts, fetal hearts and placentae due to RUPP-induced preeclampsia. Metabolomic profile of maternal hearts show reduced NAD/NADH ratio indicative of an altered redox pool (Fig. 3A), increased glucose and glycogen levels (Fig. 3C) as well as altered lipid metabolism constituents, acetate and acetyl carnitine (Fig. 3D). Furthermore, there were decreased levels of succinate (Fig. 3B) however given that the succinate/fumarate ratio was unaltered (Sham 26 ± 8 vs RUPP 15 ± 5, *P* < 0.2), the overall TCA cycle flux appears unaltered. Despite a series of changes in metabolite levels across different metabolic compartments, there is no evidence of energetic deficit or stress as creatine reserve and PCr/ATP ratio are comparable to sham controls. Furthermore, there is no change in the total adenine nucleotide (TAN) pool as ATP, ADP and AMP levels are comparable between the groups (Fig. 3A) and no alterations in the amino acid metabolism short of aspartate (Fig. 3E). Furthermore, there is evidence that preeclampsia induced maternal cardiac hypoxia as the protein levels of HIF-1α are increased compared to the sham controls (Fig. 1B) in agreement with the altered carbohydrate pool (Fig. 3C).

**Figure 4:**
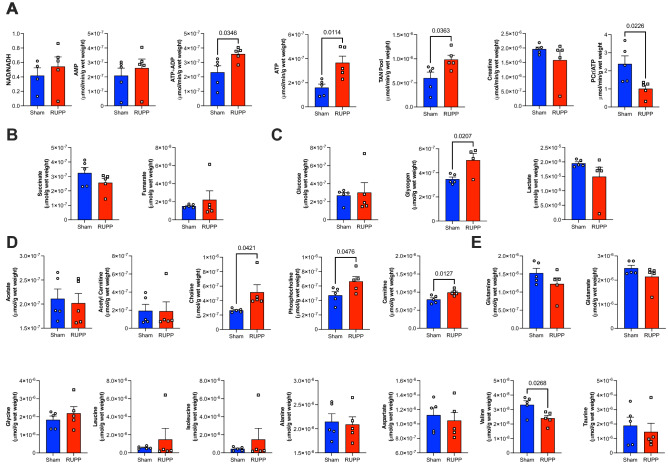
Impact of RUPP on placenta metabolism. Metabolite concentrations are grouped into (**A**) Redox and Energetics (**B**) TCA Cycle (**C**) Carbohydrate metabolism (**D**) Lipid metabolism (**E**) Amino acid metabolism. Two group comparison was carried out using the student T test (normally distributed data) or Mann-Whitney U test (non-normally distributed data: fumarate, leucine, isoleucine, lactate, glucose) TAN-total adenine nucleotide pool (AMP + ADP + ATP). n = 5/group. PCr-phosphocreatine. *P* values < 0.05 shown in individual plots.

Placentae of the RUPP dams developed various metabolic adaptations. The RUPP placenta is characterized by altered TAN pool and reduced PCr/ATP ratio energy reserve (Fig. 4A), without changes in the TCA cycle (Fig. 4B). Furthermore, there are alterations in carbohydrate, lipid and amino acid metabolism: enhanced glycogen content (Fig. 4C), increased carnitine, choline and phosphocholine levels (Fig. 4D) as well as a decrease in the levels of the amino acid valine (Fig. 4E). Reactome metabolic network analysis identified that ATP (redox and energetics) and phosphocholine (lipid metabolism) were the most influential metabolites in the placenta metabolic network (Supplementary Fig. S2).

**Figure 5:**
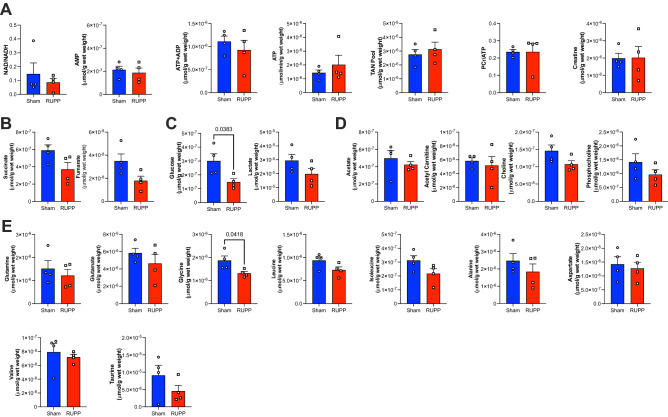
Impact of RUPP on fetal cardiac metabolism. Metabolite concentrations are grouped into (**A**) Redox and Energetics (**B**) TCA Cycle (**C**) Carbohydrate metabolism (**D**) Lipid metabolism (**E**) Amino acid metabolism. Two group comparison was carried out using the student T test (normally distributed data) or Mann–Whitney U test (non-normally distributed data: NAD/NADH, valine, ATP, Taurine, PCr/ATP) TAN-total adenine nucleotide pool (AMP + ADP + ATP). n = 4/group. PCr-phosphocreatine. *P* values < 0.05 shown in individual plots.

Remarkably, despite maternal cardiovascular remodelling including increased LV mass, cardiac hypertension^26^ and placental metabolic alterations, metabolism of the fetal hearts (Fig. 5A–E) remained unaltered as the metabolomic profile was largely comparable between RUPP and control pregnancies short of a modest reduction in glycine levels (Fig. 5E). However, of notable change is the 51% reduction in fetal myocardial glucose levels (Fig. 5C).

### Computational modelling identifies metabolic flux adaptations in the RUPP maternal hearts

**Figure 6:**
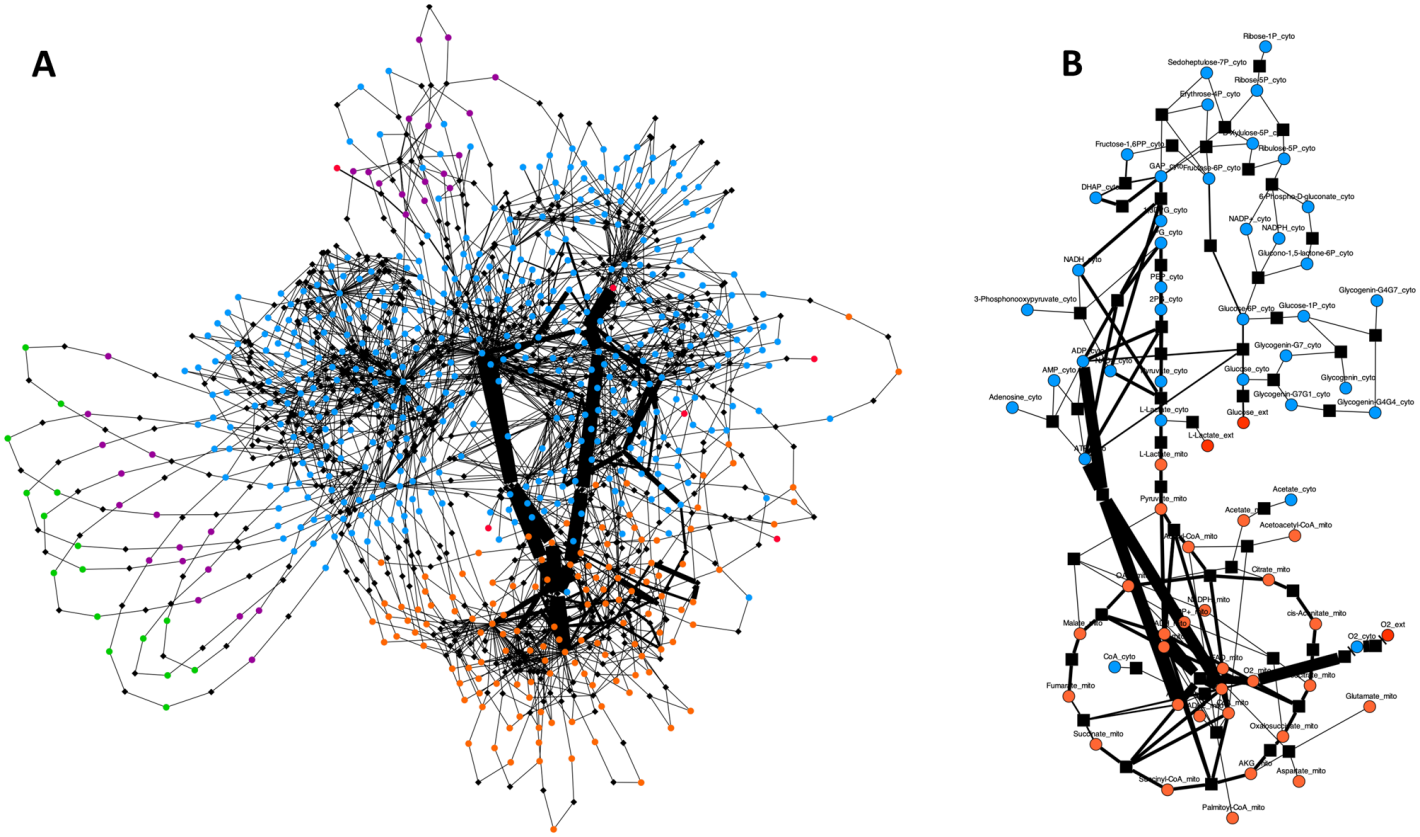
CardioNet in silico maternal heart metabolic flux changes in response to RUPP. Graph denotes estimated flux distribution in response to RUPP. The coloured nodes represent metabolites assigned to five different compartments: extracellular space, cytosol, mitochondria, microsome, lysosome. The black square nodes indicate reactions; two reactions are linked by a directed edge indicating the reaction flux. The line thickness of each edge is proportional to predicted flux rate change. Source data are provided as a Source Data file.

To assess the impact of the RUPP model of preeclampsia on maternal cardiac metabolism at a systems scale, we conducted mathematical modelling using CardioNet^31,32^. CardioNet has been successfully applied to identify limiting metabolic processes and estimate flux distributions^34–37^. We determined flux distributions using flux balance analysis (FBA), which seeks to estimate flux rates during steady-state while optimising an objective function (see Supplementary Methods for details). The optimisation problem was to maximize ATP provision within a set of constraints defined by our experimental conditions. Maternal heart metabolic data measured by ^1^H NMR spectroscopy was included into the simulations to determine which metabolic flux distributions are consistent with the maternal cardiac adaptation during preeclampsia. These simulations were conducted *without* constraining enzymatic activities; thus, the optimisation problem was defined by experimentally determined metabolite levels. Principal component analysis (PCA) (Supplementary Fig. S3A) of estimated flux distributions clearly differentiated samples according to the presence of preeclampsia. Annotation enrichment of the observed clusters and unsupervised hierarchical cluster analysis (Supplementary Fig. S3B) demonstrate extensive metabolic flux remodelling in glycolysis and lipid metabolism resulting in maintained ATP provision in the maternal heart. In silico modelling shows that RUPP hearts require overall more nutrients to maintain ATP provision and are exposed to increased ROS stress. Preeclampsia enhances glucose uptake and increases the rate of glycolysis as the key reactions involved in glycolysis were upregulated (Fig. 6) accompanied by increased lactate efflux (Fig. 6). Thus overall, Cardionet modelling indicates increased carbohydrate use. However, carbons from glycolysis are still predicted to enter the Krebs Cycle for oxidative phosphorylation. Furthermore, in silico modelling indicates that in RUPP conditions endogenous lipid pools are utilized to provide ATP. This increases oxidation of fatty acids (hexadecenoyl-CoA, stearoyl-CoA, palmitoyl-CoA, oleoyl-CoA) derived from phospholipids and is potentially responsible for the observed increase in the production of reactive oxygen species (Fig. 6).

## Discussion

In this study we have examined the impact of surgically induced reduced perfusion to the uterus in pregnant rats on the metabolism of placenta, maternal and fetal hearts. Cardiovascular alterations particularly cardiac metabolism, have not been extensively studied in this model of preeclampsia^38^. Reduced uterine perfusion leads to pathophysiological effects consistent with preeclampsia–increased fetal resorption, intrauterine growth restriction and increased haemodynamic load in maternal cardiovascular system resulting in increased heart rate, systolic, diastolic and mean arterial pressures resulting in cardiac hypertrophic enlargement^26^. Furthermore, our RUPP model is characterized by impaired placental, renal and cardiac phenotype^26^ in agreement with the other studies characterizing the RUPP model^39,40^. Given the well-established association between preeclampsia and increased risk of development of cardiovascular complications in the offspring^41^, this is an important aspect in characterizing the manifestations of pre-eclampsia. Of notice, our recently published study describes the overlapping mechanisms between preeclampsia and future cardiovascular disease with angiogenesis- and inflammatory-related pathways playing a key role^12^.

However, this is the first study that explores the maternal and fetal cardiometabolic health in the pre-clinical RUPP model of preeclampsia. Our study shows that systemic haemodynamic overload triggered by reduced uterine perfusion impacts metabolism of maternal heart and placenta. Resultant increase in HIF-1α expression corresponds to adaptive metabolic remodelling in response to RUPP^42,43^. Changes observed in RUPP maternal hearts are suggestive of adaptive metabolic remodelling as we see no evidence of energetic deficit with comparable high energy phosphates. Cardiac metabolic changes manifest as a drop in redox pool (NAD/NADH ratio) and enhanced carbohydrate metabolism in terms of uptake and storage of glucose. However, whilst the myocardial lactate concentration is comparable between the groups, our in silico modelling has shown enhanced lactate efflux in keeping with previously reported increased circulating lactate levels in RUPP models^44,45^. Furthermore, we have recently shown that altered glucose metabolism in pre-gestational or gestational diabetes results in increased risk of placental vascular dysfunction and malperfusion^46^.

Our data has shown that the RUPP model of preeclampsia induces a change in maternal myocardial substrate use concurrent with the presence of cardiac hypertrophy^47^. We observed increased levels of acetate and acetyl carnitine, potentially indicative of increased lipid utilisation. However, acetate accumulation observed in RUPP maternal hearts could also arise as the metabolic consequence of enhanced oxidative glucose metabolism and pyruvate dehydrogenase activity^48^. The RUPP metabolic profile was characterized by increased ATP demand met by increased glucose utilization but also enhanced use of endogenous lipids accompanied by increased production of reactive oxygen species (ROS). We speculate that the increased oxidation of endogenous lipids is responsible for increased ROS production. The in silico profile indicates that fatty acid oxidation is only feasible if oxygen consumption can simultaneously be increased. The model predicts that if oxygen becomes limiting, this would further increase the use of glucose by maternal RUPP hearts. The drop in the NAD^+^/NADH ratio is also indicative of the altered nicotinamide turnover, which has recently been shown to be an important hallmark of metabolic remodelling in cardiac pathologies^49^.

Expectedly, reduced uterine perfusion exhibited the strongest metabolic effect on placentae. A myriad of studies over the years have shown that the placenta is the fundamental source of fetal programming in adverse intrauterine environment and that metabolic remodelling is the incipient step in the progression to more severe forms of fetal growth restriction^50^. Reduced tissue perfusion normally triggers a range of responses including increased angiogenesis and erythropoiesis in order to improve O_2_ availability needed for cellular survival under hypoxic conditions. In both human and animal placentas, hypoxia-triggered responses result in increased density of villous capillaries and elevated secretion of angiogenic and hematopoietic factors (ie. VEGF, erythropoietin)^51–56^. However, as our previous work has shown, preeclampsia is characterized by an anti-angiogenic phenotype driven by the upregulation in FKBPL expression or increase in sFlt-1, which would disrupt this primary hypoxia defence mechanism triggered by reduced perfusion^57,58^.

Increased ATP levels driving an increase in the TAN pool are indicative of altered placental ATP homeostasis driven by reduced perfusion and resultant hypoxia as evidenced by increased HIF-1α expression. In non-placenta tissue including hearts this would be a rare finding as ATP levels are tightly regulated (in part via the Randle cycle) to avoid energetic waste. This observation could potentially have two implications. First, RUPP placentae with an anti-angiogenic phenotype^17^ and altered HIF-1α could lead to reduced energy demand by reducing the activity of ATP utilisers, thus reducing ATP use. This response in placental biology is broadly defined as “demand reduction” and consists of the attenuation of energy-requiring processes, non-essential to cellular survival, including gross energy demands such as fetal movement^59^. At the tissue level the inhibition of transcription, translation and related processes is dependent on protein synthesis, such as cellular growth and proliferation^60^. Furthermore, this interpretation would be entirely consistent with the traditional observation that hypoxia triggers adaptation to augment the ATP supply including via non-oxidative pathways. In the placenta this process has been demonstrated most clearly by an increase in glucose uptake and consumption as well as other key elements of the glycolytic metabolism^50,61–65^. Reduction in oxidative pathways, specifically a shift towards carbohydrate metabolism of RUPP placentae, would also be supported by the accumulation of carnitine due to altered fatty acid use.

Furthermore, we also observe changes in placental choline and phosphocholine levels, indicative of altered and enhanced nutrient transport in response to restricted vascularization and perfusion. Choline is an important bioactive micronutrient often grouped with the B vitamins. Placental transport of choline from the maternal circulation is largely mediated by the choline transporter-like protein 1 (CTL1)^66^. Within the placenta, choline and its metabolites perform crucial roles in placental and foetal development, especially the development of the brain. Enhanced choline uptake from maternal circulation, which would lead to enhanced levels in the placenta, would help to maintain both essential and non-essential amino acids for fetal development as well as enhance glycogen synthesis in the placenta as the additional source of energy^67^.

Thus, enhanced choline and its metabolite phosphocholine levels would also explain the increased glycogen content we observed in RUPP placentae. Whilst the metabolomic profile of amino acids was mostly unaffected by RUPP, levels of valine were markedly reduced suggesting enhanced fetal uptake and oxidation^68^.

Despite the series of metabolic adaptations in maternal hearts and placentae we observed in our study, these were unable to prevent intrauterine growth restrictions and fetal loss as RUPP dams had higher fetal resorption rate in agreement with both human preeclampsia and animal model outcomes. Although surviving embryos were smaller overall^26^, there is no evidence of extensive cardiometabolic remodelling. However, whilst there may not be many metabolite levels altered in the hearts of RUPP pregnancy fetuses, there is a marked reduction in the level of the most critical metabolite, glucose. Unlike adult hearts, fetal hearts rely on glucose as the main source of ATP^47^. Glucose is the principal energy substrate for the placenta and the fetus and is essential for normal fetal metabolism and growth. However, given that there are no alterations in fetal heart energy reserve, redox pool, lipid metabolism, TCA cycle intermediates short of a reduction in glycine levels, the observed reduction in glucose levels is most likely the result of reduced transport across RUPP placenta rather than due to extensive metabolic rewiring^69^.

While this study reports the response of maternal and fetal heart tissues to preeclampsia-driven stress, future studies may help to further evaluate the underlying mechanisms that regulate the effects of preeclampsia on fetal cardiac function. This should include the effects of comorbidities often present with preeclampsia such as obesity and type 2 diabetes. In our RUPP model, we demonstrate that preeclampsia leads to fetal loss, cardiac remodelling and dysfunction showing likely signs of diastolic dysfunction^26^. Furthermore, we demonstrate, for the first time that preeclampsia drives an adaptive metabolic remodelling axis encompassing maternal heart and placenta. However, these adaptations are unable to prevent overall intrauterine growth restriction and fetal loss in RUPP rats despite largely unaltered fetal heart metabolism. We cannot however exclude the possibility that metabolic adaptation of the maternal tissues drives enhanced fetal resorption in order to conserve substrates and O_2_ for the remaining fetuses. Furthermore, placenta and maternal heart adaptations could be protecting the metabolism of the developing fetal hearts. Nevertheless, long-term consequences of this adaptive maternal cardiovascular changes need to be investigated further.

Potential metabolic mechanisms identified in this study could inform future studies intending to provide better understanding of the increased risk of cardiovascular disease in women affected by preeclampsia. This could also provide improved prevention strategies for reducing the risk of future cardiovascular complications following preeclampsia.

## Supplementary Information

Supplementary Information.

## Data Availability

All data supporting the results presented herein are available from the corresponding author upon reasonable request. Cardionet modelling data are provided in a Supplementary File. Database availability: Cardionet MODEL1212040000;
