# Supplementary material for: Impact of reduced uterine perfusion pressure model of preeclampsia on metabolism of placenta, maternal and fetal hearts
Source: Sci Rep. 2022 Jan 21;12:1111. doi: 10.1038/s41598-022-05120-2 (PMC8782944; doi:10.1038/s41598-022-05120-2)
Supplement: Supplementary file 1 — Supplementary Information. [file 41598_2022_5120_MOESM1_ESM.docx]

**Supplementary Material**

**Methods**

**Real-time quantitative polymerase chain reaction (RT-qPCR)**

**Supplementary Table S1.** Nucleotide sequence of primers used in RT-qPCR

| **Name of primer** | **Sequence (5’-3’)** |
| --- | --- |
| β-actin (sense) | AAGACCTCTATGCCAACAC |
| β-actin (antisense) | TGATCTTCATGGTGCTAGG |
| Galectin-3 (sense) | ACCATTCAAAATACAGGTCC |
| Galectin-3 (antisense) | TGGTTATACTGCAACAGATG |

**Mathematical modeling of the maternal myocardial metabolic adaptations to RUPP**

*Cardionet in silico analysis*

*In silico* simulations were performed using the metabolic network of the cardiomyocyte CardioNet^1-3^. Mathematical modeling has previously been used to study the dynamics of cardiac metabolism in response to stress,**^3-5^** and CardioNet has been successfully applied to identify limiting metabolic processes and estimate flux distributions.**^1,5^** Flux balance analysis (FBA) allows the estimation of flux rates in a cellular model based on metabolic constraints that are defined by the extracellular environment (e.g. oxygen and nutrient supply), cellular demands (e.g. proliferation, contraction) and tissue type. This modeling approach combines biochemical network models with optimality problems, which describe different cost or benefit functions and allow us to include experimental data, for example metabolite levels, enzyme levels or flux rates. The advantage of flux balance analysis is that it considers system-wide effects of processes and allows us to assess metabolic limitations in an unbiased approach. We applied flux balance analysis to identify which reactions are involved in myocardial metabolic adaptations to RUPP.

Metabolic flux distributions were calculated using constrained based modeling. To calculate flux rate changes (*v_i_*), we constrained the model for each metabolite using experimentally determined metabolite concentrations (^1^H NMR) to maximize cardiac work reflected by ATP hydrolysis (v_ATPase_). Simulations were run with boundary conditions reflecting the circulating metabolite composition based on previously reported values and common metabolites present in plasma^6-8^,^9^. Based on these constrains we first determined flux distributions ($v_{m}$) under sham operation (control) conditions. We then calculated fold-changes (FC) for experimentally measured metabolite concentrations between sham controls and RUPP groups, and used these fold-changes to further constrain fluxes ($v_{m}$) for the synthesis and/or degradation of intracellular metabolites. We included fold-changes (FC) based on the assumption that changes in metabolite concentrations under experimental conditions are accompanied by a proportional increase or decrease in the respective flux for the metabolite pool. By using metabolite level changes (fold changes) to estimate flux rate changes (v_FC_), we imply that the altered steady-state concentrations of metabolites are reflected in the newly evolved flux state and potentially limit metabolic functions.

The following flux balance analysis was applied to identify steady-state flux distributions that agree with applied substrate uptake and release rates, and changes in metabolite pools:

$$\max v_{ATPase}$$

subject to

$$S\cdot v=0,$$

$$v_{i}^{(-)}\leq v_{i}\leq v_{i}^{\left( + \right)},$$

$$L_{j}^{(-)}\leq v_{j}\leq L_{j}^{\left( + \right)} \left( j=j_{1}, j_{2}, \ldots\right),$$

$$v_{m}\leq{FC}_{m} \cdot v_{m}^{0} \left( m=m_{1}, m_{2}, \ldots\right),$$

where $v_{i}$ denotes the flux rate change through reaction $i$, $v_{j}$ denotes the measured uptake or secretion rate through reaction $j$, *S* is the stoichiometric matrix, and $v_{i}^{(-)}$ and $v_{i}^{(\mp)}$ are flux constraints. The GUROBI LP solver was used to find the solution to the FBA problems.^10^ The logarithm of the metabolic flux rate values is presented in the form of heatmaps. Metabolic reactions are clustered according to their association to metabolic pathways and plot colours indicate estimated flux rates for each metabolic reaction. All reactions and their metabolic subsystems, classified in the Kyoto Encyclopedia of Genes and Genomes database^11^.

**Results**

**Supplementary Figure S1. Galectin 3 expression in maternal heart and placenta**

Gal-3 mRNA expression normalised to a housekeeping gene (β-actin)

**Supplementary Figure S2. Metabolite co-regulation network of RUPP placenta**

Only the metabolites with pronounced pairwise correlation (Pearson correlation coefficient |r| >0.7) were shown in this plot, where each node represented an individual metabolite.

To assist visualisation, node colour was encoded by the function of corresponding metabolite; edge colour and length indicated Pearson correlation coefficient r, thus the closer the more correlated and vice versa; node size was proportional to the epigenvector centrality (the importance of a metabolite within the network).

**B**

**Supplementary Figure S3. Impact of RUPP on maternal cardiac metabolic profile**

**A)** Principal component analysis (PCA) of CardioNet simulation for Sham and RUPP maternal cardiac metabolomic profile. Ellipses indicate 95% confidence interval. The first two components of the PCA explain 71.4% of the variability. Sample segregated into control Sham and RUPP maternal cardiac metabolism.

**B)** Unsupervised hierarchical clustering of estimated z-scored flux rate changes reveals metabolic adaptation in response to RUPP. Heat maps summarize results for reactions in the Krebs cycle, OXPHOS, and glucose metabolism. Flux distributions were calculated by Flux balance analysis (FBA) using the mammalian network of cardiac metabolism, CardioNet. Z-scores were calculated to visualize how many standard deviations an estimated flux rate is away from the mean across all experimental groups. The z-score describes the distance from the mean for a given flux rate as a function of the standard deviation.

For example, a z-score equal to 1 represents a flux for a given experimental group that is 1 standard deviation greater than the mean across all experimental groups.

The colour scale indicates the degree to which estimated flux rate changes are predicted to be respectively lower or higher in response to RUPP. Source data are provided in Supplementary Data file.

**References**

1 Aksentijevic, D. *et al.* Cardiac dysfunction and peri-weaning mortality in malonyl-coenzyme A decarboxylase (MCD) knockout mice as a consequence of restricting substrate plasticity. *J Mol Cell Cardiol* **75**, 76-87, doi:10.1016/j.yjmcc.2014.07.008 (2014).

2 Karlstaedt, A. *et al.* Oncometabolite d-2-hydroxyglutarate impairs alpha-ketoglutarate dehydrogenase and contractile function in rodent heart. *Proc Natl Acad Sci U S A* **113**, 10436-10441, doi:10.1073/pnas.1601650113 (2016).

3 Karlstadt, A. *et al.* CardioNet: a human metabolic network suited for the study of cardiomyocyte metabolism. *BMC Syst Biol* **6**, 114, doi:10.1186/1752-0509-6-114 (2012).

4 Ch'en, F. F., Vaughan-Jones, R. D., Clarke, K. & Noble, D. Modelling myocardial ischaemia and reperfusion. *Prog Biophys Mol Biol* **69**, 515-538 (1998).

5 Cortassa, S. *et al.* A computational model integrating electrophysiology, contraction, and mitochondrial bioenergetics in the ventricular myocyte. *Biophys J* **91**, 1564-1589, doi:10.1529/biophysj.105.076174 (2006).

6 Chua, B. H., Siehl, D. L. & Morgan, H. E. A role for leucine in regulation of protein turnover in working rat hearts. *The American journal of physiology* **239**, E510-514 (1980).

7 Goodwin, G. W., Ahmad, F., Doenst, T. & Taegtmeyer, H. Energy provision from glycogen, glucose, and fatty acids on adrenergic stimulation of isolated working rat hearts. *The American journal of physiology* **274**, H1239-1247 (1998).

8 Swanton, E. M. & Saggerson, E. D. Effects of adrenaline on triacylglycerol synthesis and turnover in ventricular myocytes from adult rats. *Biochem J* **328 ( Pt 3)**, 913-922 (1997).

9 Karlstädt, A. *et al.* CardioNet: a human metabolic network suited for the study of cardiomyocyte metabolism. *BMC Syst Biol* **6**, 114 (2012).

10 Gurobi Optimization, L. Gurobi Optimizer Reference Manual. (2021).

11 Ogata, H. *et al.* KEGG: Kyoto Encyclopedia of Genes and Genomes. *Nucleic acids research* **27**, 29-34 (1999).
